# Graph Clustering with High-Order Contrastive Learning

**DOI:** 10.3390/e25101432

**Published:** 2023-10-10

**Authors:** Wang Li, En Zhu, Siwei Wang, Xifeng Guo

**Affiliations:** 1 School of Computer Science, National University of Defense Technology, Changsha 410000, China; leon20080518@163.com (W.L.); wangsiwei13@nudt.edu.cn (S.W.); 2 School of Cyberspace Science, Dongguan University of Technology, Dongguan 523808, China

**Keywords:** graph clustering, unsupervised learning, contrastive learning, augmentation

## Abstract

Graph clustering is a fundamental and challenging task in unsupervised learning. It has achieved great progress due to contrastive learning. However, we find that there are two problems that need to be addressed: (1) The augmentations in most graph contrastive clustering methods are manual, which can result in semantic drift. (2) Contrastive learning is usually implemented on the feature level, ignoring the structure level, which can lead to sub-optimal performance. In this work, we propose a method termed Graph Clustering with High-Order Contrastive Learning (GCHCL) to solve these problems. First, we construct two views by Laplacian smoothing raw features with different normalizations and design a structure alignment loss to force these two views to be mapped into the same space. Second, we build a contrastive similarity matrix with two structure-based similarity matrices and force it to align with an identity matrix. In this way, our designed contrastive learning encompasses a larger neighborhood, enabling our model to learn clustering-friendly embeddings without the need for an extra clustering module. In addition, our model can be trained on a large dataset. Extensive experiments on five datasets validate the effectiveness of our model. For example, compared to the second-best baselines on four small and medium datasets, our model achieved an average improvement of 3% in accuracy. For the largest dataset, our model achieved an accuracy score of 81.92%, whereas the compared baselines encountered out-of-memory issues.

## 1. Introduction

As a powerful tool, the Graph Neural Network (GNN) has been designed to deal with graph data such as social networks, knowledge graphs, citation networks, etc. The invention of the GNN has greatly facilitated graph-related tasks such as graph classification [1,2,3], neural machine translation [4,5], relation extraction [6,7], relational reasoning [8,9], and graph clustering [10,11,12]. Unlike traditional clustering methods such as K-means, GNN-based graph clustering models use deep neural networks for representation learning before clustering. Adaptive graph convolution (AGC) [11] is a method that can adaptively choose its neighborhood over various graphs. A deep attentional embedded graph clustering model (DAEGC) [13] can learn to aggregate neighbors by calculating their importance. The adversarially regularized graph autoencoder (ARGV) [14] introduces adversarial regularization to learn and improve the robustness of representations. The work on attributed graph embedding (AGE) [15] proposed a Laplacian filtering mechanism that can effectively denoise features. The deep fusion clustering network (DFCN) [16] is a hybrid method that integrates embeddings from autoencoder (AE) [17] and graph autoencoder (GAE) [18] modules for representation learning.

Recently, there has been growing interest in contrastive learning. Applying contrastive learning to deep graph clustering has become more common than before. The principle of contrastive learning is to bring similar or positive sample pairs closer and push dissimilar or negative sample pairs further away from each other. Graph clustering is a fundamental but challenging task in graph analysis. The contrastive multi-view representation learning method (MVGRL) [19] has achieved its best performance by contrasting the embeddings of the nodes and sampled sub-graphs. Specifically, it constructs an extra diffusion graph for contrastive learning. The node embeddings from one view are contrasted with the sub-graph embeddings from the other. The method determines which nodes and sub-graphs belong to the positive pair and which belong to the negative pair. The self-consistent contrastive attributed graph clustering method (SCAGC) [20] can maintain the consistency between the learned representation and cluster structure by performing contrastive learning between clusters and between nodes with the guidance of clustering results. Inspired by the deep graph infomax method (DGI) [21], the community detection-oriented deep graph infomax method (CommDGI) [22] introduced a community mutual information loss to capture the community structural information for nodes.

**Figure 1:**
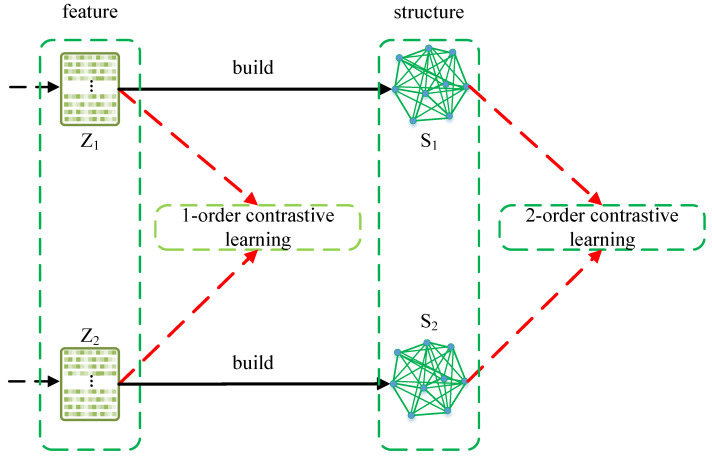
First-order contrastive learning and second-order contrastive learning. $Z_{1}$ and $Z_{2}$ denote the features, and $S_{1}$ and $S_{2}$ are the similarity matrices built by $Z_{1}$ and $Z_{2}$.

Although promising performance has been achieved, there still exist problems that need to be addressed. Firstly, in existing methods, manual augmentation such as feature masks and edge drops can result in semantic drift, which leads to sub-optimal performance. Secondly, most of the methods perform contrastive learning on feature-based (first-order) contrastive similarity, ignoring structure-based (second-order) contrastive similarity, which leads to sub-optimal performance. Figure 1 shows the difference between first-order contrastive learning and second-order contrastive learning.

To solve the above-mentioned problems, we propose a contrastive graph clustering method termed Graph Clustering with High-Order Contrastive Learning. To address the first problem, we build two views by performing Laplacian smoothing with different normalizations on the same features. We build two similarity matrices with features. Each element in the similarity matrices denotes the similarity between nodes. We argue that the corresponding embeddings can be mapped into the same space using the alignment loss between the similarity matrices. To address the second problem, we build a contrastive similarity matrix using the similarity matrices. Inspired by [23], we perform contrastive learning by minimizing the loss between the contrastive similarity matrix and an identity matrix. In this way, our model can implement contrastive learning at the structure level. Meanwhile, the contrastive similarity matrix is built using the feature-based similarity matrix, and contrastive learning can also be assumed to be at the feature level to some degree. Furthermore, we can learn clustering-friendly representations naturally without the manual sampling that is applied in most contrastive methods and we need no extra clustering algorithms for training. Moreover, our method can be trained on large datasets. The key contributions of this paper are as follows:

- Without any manual augmentations, we use two different Laplacian smoothing methods to build two views for contrastive learning and design an alignment loss to force the learned embeddings to map into the same space.
- We design a novel structure-based contrastive loss without a sampling phase. By contrasting two similarity matrices, our model can learn clustering-friendly representations. It is worth noting that our model can also be applied to large-scale datasets.
- Extensive experiments on five open datasets validate the effectiveness of our model.

## 2. Related Works

In this paper, we roughly divide deep graph clustering models into two kinds—reconstructive and contrastive—and we introduce them in the following subsections. The definitions of the acronyms used here can be found in Appendix A.2.

### 2.1. Deep Reconstructive Graph Clustering

Reconstructing graphs or features is a basic learning paradigm in many deep clustering graphs. It can be divided into three categories: reconstruction only, adversarial regularization, and hybrid. The graph autoencoder (GAE) [18] is a basic model that is often introduced in graph clustering models as the framework. DAEGC [13] and MGAE [12] are models that are trained by reconstructing the given structure or raw features. ARGV and AGAE [10,14] can improve the robustness of the learned representations by introducing adversarial regularization. SDCN, AGCN, and DFCN [16,24,25] are typical hybrid models. SDCN can alleviate over-smoothness by integrating the representations from the AE and GCN. Based on SDCN, AGCN includes an adaptive fusion mechanism to improve the graph representations. DFCN includes a triple loss function to improve the robustness of the graph representations. All these models need an extra clustering module to learn clustering-friendly representations. Our model can naturally learn the clustering-friendly representations through high-order contrastive learning.

### 2.2. Deep Contrastive Graph Clustering

The effectiveness of contrastive learning has been widely validated. Applying contrastive learning to the deep graph clustering model has recently become a trend. The aim of Sublime [26] is to improve the anchor graph by constructing a learned auxiliary graph. By contrasting the node embeddings of the anchor graph and the learned graph, Sublime can reduce the impact of noisy connections or missing connections. Inspired by [23], DCRN [27] is used to perform feature decorrelating in two different ways, but it still needs a clustering module to learn clustering-friendly representations. GDCL [28] employs a debiased method to choose negative samples. Specifically, it defines the nodes and their augmented ones as the positive pairs and defines the node pairs with different pseudo-labels as the negative pairs. This way, it alleviates the impact of false-negative samples. SAIL [29] utilized self-distillation to maintain distribution consistency between low-layer node embeddings and high-layer node features and alleviate the problem of smoothness. The idea behind AFGRL [30] is that augmentation on graphs is difficult to design. Therefore, it employs an augmentation-free method by combining KNN, K-means, and the adjacency matrix to capture the local and global similarities of nodes, and the obtained guidance can help contrastive learning. AutoSSL [31] adaptively combines different pre-text tasks to improve graph representation learning. These contrastive models are characterized by manual augmentation, sampling positive and negative pairs, and first-order contrastive learning. Manual augmentation can result in semantic drift, the sampling strategy needs an extra clustering-oriented module to define the positive and negative pairs, and first-order contrastive learning can only learn clustering-friendly representations from the feature perspective, ignoring the structure perspective. Our model can effectively alleviate these issues.

## 3. Proposed Method

**Figure 2:**
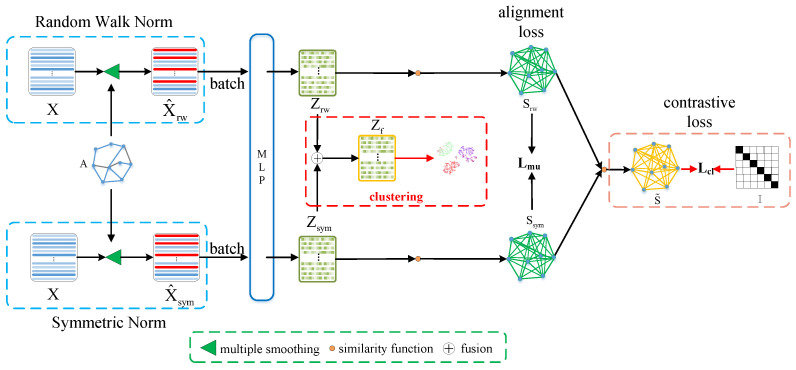
The overall framework of the GCHCL model.

In this section, we propose an algorithm for the Graph Clustering with High-Order Contrastive Learning model. The entire framework of our model is shown in Figure 2. Below, we describe the proposed GCHCL model.

### 3.1. Problem Definition

In this paper, $V=\{v_{1},v_{2},\ldots ,v_{n}\}$ is a set of *N* nodes, and *E* denotes an edge set. Given an undirected graph G=(X,A), $X\in R^{n\times d}$ denotes the attribute matrix, and $A={\left(a_{ij}\right)}_{n\times n}$ denotes the given adjacency matrix. In the adjacency matrix *A*, $a_{ij}\in 0,1$. $a_{ij}=1$ indicates an explicit connection between $v_{i}$ and $v_{j}$. Otherwise, there exists no direct connection between them. We let $D=diag\left(d_{1},d_{2},\ldots ,d_{N}\right)\in R^{n\times n}$ be the degree matrix. $d_{i}$ indicates the ith row in *D* and $d_{i}=\sum_{j=1}^{n}a_{ij}$. The Laplacian matrix of the graph is built as L=D−A. Details about the notations used are shown in Table 1.

### 3.2. Double Laplacian Smoothing

In several works, Laplacian smoothing has been proven to be effective in alleviating the impact of high-frequency noise [15,32]. In [15], the GCN was decoupled into a graph filter and a linear transformation, and it was demonstrated that the decoupled GCN could achieve the same or even better performance in representation learning compared to the GCN. Generally, the features are convolved by the Laplacian matrix to avoid gradient explosion during training; therefore, the Laplacian matrix needs to be normalized. There are two types of normalization: random walk normalization and symmetric normalization. During the aggregation step, the random walk-normalized Laplacian matrix treats the neighbors equally. However, the symmetric-normalized Laplacian matrix considers both the degree of the target node and its neighbors’ degrees. The larger the degree of the neighbor, the smaller its contribution to the aggregation. The random walk-normalized Laplacian matrix is constructed as follows:

(1) $$L_{rw}=I-{\hat{D}}^{-1}\hat{A}$$

The symmetric normalized matrix is constructed as follows:

(2) $$L_{sym}=I-{\hat{D}}^{-\frac{1}{2}}\hat{A}{\hat{D}}^{-\frac{1}{2}}$$

where $\hat{A}=A+I$, $\hat{D}$ is the degree matrix of $\hat{A}$. With these two types of normalized Laplacian matrices, we construct two different views for the same feature matrix, as follows:

(3) $$H_{sym}=I-L_{sym}$$

(4) $$X_{sym}={H_{sym}}^{t}X$$

(5) $$H_{rw}=I-L_{rw}$$

(6) $$X_{rw}={H_{rw}}^{t}X$$

where *t* is the power of the filter operation.

### 3.3. Structure Alignment

After randomly sampling batches of nodes, we construct two different views for each batch of nodes without augmentation and force them to be mapped into the same space. For simplicity, we use a simple linear transformation as the encoder. First, we sample nodes with an assigned batch size:

(7) $$X_{rw}^{b}=Sample\left(X_{rw}\right)$$

(8) $$X_{sym}^{b}=Sample\left(X_{sym}\right)$$

where Sample is a random sample operation, and *b* is the assigned batch size. $X_{rw}^{b},X_{sym}^{b}\in R^{b\times f}$. After sampling, nodes are input to the encoder in batches, as follows:

(9) $$Z_{rw}=Encoder\left(X_{rw}^{b}\right)$$

(10) $$Z_{sym}=Encoder\left(X_{sym}^{b}\right)$$

(11) $${\hat{Z}}_{rw}=\frac{Z_{rw}}{∥Z_{rw}∥}$$

(12) $${\hat{Z}}_{sym}=\frac{Z_{sym}}{∥Z_{sym}∥}$$

(13) $$Z_{f}=\frac{1}{2}\left({\hat{Z}}_{sym}+{\hat{Z}}_{rw}\right)$$

To force the two views of sampled attributes to be mapped into the same embedding space, we design a structure-aligning loss. Specifically, we build two similarity matrices using the output of the encoder. By minimizing the alignment loss between the two similarity matrices, we can map the embeddings to the same space and maintain the consistency of their distribution. The processing is as follows:

(14) $$S_{rw}=<{\hat{Z}}_{rw},{\hat{Z}}_{rw}>$$

(15) $$S_{sym}=<{\hat{Z}}_{sym},{\hat{Z}}_{sym}>$$

(16) $$L_{sl}=\frac{1}{2b}{∥{\hat{S}}_{rw}-{\hat{S}}_{sym}∥}_{F}^{2}$$

where <> denotes the operation of the inner product, and Sim denotes a similar metric function such as a cosine function.

### 3.4. High-Order Structure Contrastive Learning

Instead of performing contrastive learning on the first-order contrastive similarity, we perform contrastive learning on the second-order contrastive similarity. Compared to contrastive learning on the first-order similarity, contrastive learning on the second-order similarity can provide a wider view. In a structure-based contrastive similarity matrix, ${\hat{S}}_{ij}$ denotes the structural similarity of node *i* and node *j*. Moreover, structure-based contrastive learning is based on the similarity matrix; therefore, it also implies a similarity of features. Inspired by [23], we implement contrastive learning as follows:

(17) $$\tilde{S}=Sim\left(S_{rw},S_{sym}\right)$$

(18) $$L_{cl}=\frac{1}{2b}{∥\tilde{S}-I∥}_{F}^{2}$$

where $\tilde{S}$ is the structure-based contrastive similarity matrix.

### 3.5. Joint Optimization

On the one hand, the alignment of the structure similarity matrices can force the embeddings to map into the same space. On the other hand, contrastive learning on the similarity matrices can naturally benefit the clustering task. By jointly optimizing these two objective functions, we train our model as follows:

(19) $$\begin{array}{cc} L & =L_{sl}+L_{cl}\\ \; & =\frac{1}{2b}∥S_{rw}-S_{sym}{∥}_{F}^{2}+\frac{1}{2b}{∥\tilde{S}-I∥}_{F}^{2} \end{array}$$

The details of the training process are shown in Algorithm 1.
**Algorithm 1** Graph Clustering with High-Order Contrastive Learning**Input**: Attribute matrix *X*, adjacency matrix *A*, training iteration *T*, identity matrix *I*, number of clusters *K*, number of nodes *n*, hyperparameters t,b1:Build two kinds of normalized Laplacian matrices using (1) and (2)2:Build two views of the filtered attributes using (3)–(6)3:**for** i=1 to *T* **do**4:   **for** j=1 to (nmodb) **do**5:       Randomly sample *b* nodes from each view using (7) and (8)6:       Generate the embeddings ${\hat{Z}}_{rw}$ and ${\hat{Z}}_{sym}$ using (9)–(12)7:       Build the similarity matrix using (14) and (15)8:       Build the contrastive similarity matrix using (17)9:       Calculate the alignment loss of the similarity matrices using (16)10:     Calculate the contrastive loss of the contrastive similarity matrix and an identity matrix using (18)11:     Update the whole framework by minimizing (19)12:   **end for**13:**end for**14:Obtain the fusion embeddings $Z_{f}$ using (13)15:Perform K-means clustering on $Z_{f}$  **Output**: The clustering result *O*

### 3.6. Complexity Analysis

In this paper, we denote *d* as the dimension of the encoder, *b* as the sampled size of the nodes, and *f* as the dimension of the raw features. The computational complexity of our model is $O(bfd+b^{2}d+b^{3})$. Specifically, the complexity of the encoder is O(bfd), the complexity of constructing a similarity matrix is $O(b^{2}d)$, and the complexity of constructing the contrastive similarity matrix is $O(b^{3})$. Thus, the entire computational complexity of the proposed model is $O(bfd+b^{2}d+b^{3})$. The complexity of our model is dominated by the scale of the batch size.

## 4. Experiment

### 4.1. Dataset

We conducted extensive experiments on five widely used benchmark datasets: Cora, Dblp, Amap, Corafull, and Reddit. More details can be found in Table 2.

- **Cora** [18] is a citation dataset. Each node denotes a machine learning paper, and each edge denotes the citation relationship between two papers. The papers within it are divided into seven classes: case-based, genetic algorithms, neural networks, probabilistic methods, reinforcement learning, rule learning, and theory. Each node’s feature is represented by a 0, 1 vector. Each dimension is a keyword from a specific vocabulary.
- **Dblp** [24] is a cooperative network. The authors are categorized into four classes: database, data mining, machine learning, and information retrieval. Each edge represents a collaborative relationship between authors. The node features consist of elements from a bag-of-words method represented by keywords.
- **Amap** [33] is a co-purchase graph dataset. Each node denotes a type of good, and each edge denotes the corresponding goods that are often purchased together. These nodes are divided into eight classes according to the category of the goods.
- **Corafull** [33] is similar to Cora but is larger, and the papers within it are divided into 70 classes.
- **Reddit** [1] is constructed from Reddit posts from September 2014. Each node denotes a post, and each edge denotes two posts commented on by the same user. The posts are divided into 41 classes. The node features are the average of 300-dimensional GloVe word vectors associated with the content of the posts, including the title, comments, score, and number of comments.

### 4.2. Experimental Setup

All experiments were run on a computer with a GeForce RTX 1080Ti GPU, 64 G RAM, and Pytorch 1.8.1. We set the maximum number of iterations for training to 100 for all datasets. We optimized our model using the Adam optimizer. When the training process stopped, we ran the K-means clustering algorithms on the learned embeddings. To reduce the impact of randomness, we repeated each experiment 10 times and report the average results.

### 4.3. Parameter Setting

In our model, we used a single-layer MLP as the encoder. The dimension of the output was 100 for Reddit and 500 for the other datasets. For simplicity, we used no activation function except for a linear transformation. In our model, instead of inputting the whole feature matrix for training, we performed the training in batches with an assigned batch size. Specifically, we denoted *b* as the batch size. For Amap and Reddit, we set b=256; for Cora and Corafull, we set b=512; and the batch size for Dblp was 1024. Regarding the compared baselines, we utilized the settings specified in their respective papers. The details of the hyperparameters are shown in Table 3.

### 4.4. Metrics

The clustering performance was evaluated on four widely used metrics: ACC (Accuracy) [34], NMI (Normalized Mutual Information) [35], ARI (Average Rank index) [36], and F1 (macro-F1 score) [37].

### 4.5. Performance Comparison

In our experiments, we compared our model to 14 methods on five benchmark datasets. Specifically, K-means is the classic clustering algorithm. GAE, VGAE, MGAE, and DAEGC [12,13,18] are reconstructive learning methods. ARGE and ARVGE [14] are adversarial regularization methods. AGCN, SDCN, and DFCN [16,24,25] are hybrid methods. SCAGC, GDCL, MVGRL, AutoSSL, and Sublime [19,20,26,28,31] are contrastive learning-based methods. Details on the performance comparison can be found in Table 4, Table 5, Table 6, Table 7 and Table 8. The best results are marked in **bold**. From the information in these tables, we can make the following observations:

- The proposed model achieved the best performance in most cases. For example, on the Amap dataset, our model achieved ACC, NMI, ARI, and F1 scores of 79.18%, 70.37%, 62.22%, and 72.93%, respectively, We observed relative improvements of 1.1%, 1.5%, 2.7%, and 5.3% over the second-best baseline on the Cora, Dblp, Amap, and Corafull datasets.
- K-means performed clustering directly on the raw features and could, to some degree, indicate the quality of the attributes of the dataset. As can be seen, the attributes of the Cora dataset demonstrated the highest quality for clustering. The baselines from GAE to DFCN were classical deep graph clustering models and were mostly trained by reconstructing the raw features or the given graphs. GAE, VGAE, MGAE, ARGE, ARVGE, AGCN, and DAEGC were sub-optimal compared to our model because they only used a single view for embeddings, which had a limitation in providing diverse features for representation learning. SDCN and DFCN learned the representations through a cross-module approach, enriching the information for learning. The reason our model outperformed SDCN and DFCN was that they heavily relied on the provided graph, which could not fully reveal the complete connections between nodes and may have misled representation learning. The utilization of a similarity matrix in our model can greatly alleviate this.
- The baselines from SCAGC to Sublime are graph clustering models based on contrastive learning. All of them implemented contrastive learning at the feature level, which could not effectively capture the neighborhood of each node, an important aspect for clustering tasks. Our model directly performed contrastive learning at the structural level. This allows the contrastive learning in our model to facilitate the clustering task more effectively.
- On the Reddit dataset, most of the baselines struggled with the training cost, leading to OOM (out-of-memory) issues. There are two reasons for this: (1) they usually input the whole dataset into the model during training, and (2) the entire adjacency matrix consistently participated during training. In our model, we input batches of features into the model instead of the whole feature matrix, which greatly reduced the computations.

### 4.6. Ablation Study

We performed an ablation study from two perspectives: (1) To validate the effectiveness of high-order contrastive learning, we implemented two experiments, one on first-order contrastive learning and one on second-order contrastive learning. (2) To assess the effectiveness of each component in our model, we conducted experiments by individually removing the structure alignment and contrastive learning.

In Table 9, we can observe that the contrastive learning on the first-order similarity matrix consistently underperformed compared to the second-order similarity matrix. This is because first-order contrastive learning is based on feature similarity, which may lead to representation bias. However, second-order contrastive learning is based on neighborhood similarity, which can alleviate this bias. In addition, compared to first-order contrastive learning, second-order contrastive learning can learn clustering-oriented representations more effectively.

In Table 10, we can observe that each component in our model contributed to the performance. Specifically, when we removed the contrastive part, the performance decreased significantly on all datasets. This is because without CL, the representation bias impacted the performance across all datasets. When SA was omitted, the impact on the performance for the Cora, Dblp, Amap, and Corafull datasets was minimal, but for the Reddit dataset, it was significant. This was because CL carried a risk of reducing useful relationships, which could harm performance, but SA could preserve these relationships, alleviating this issue. The model conducted graph convolution five times on Reddit, and no more than three times on the other datasets. By aggregating more neighbors, the number of similar nodes to the target one increased in the embedding space. When the model performed contrastive learning on the similarity matrices of the Reddit dataset, it reduced more useful relationships compared to the other datasets. Therefore, the performance decreased more on the Reddit dataset compared to the others.

### 4.7. Hyperparameter Analysis

In this paper, we introduced two hyperparameters *b* and *t*. *b* denotes the batch size of the input features, and *t* is used to control the power of the Laplacian smoothing before training.

**Figure 3:**
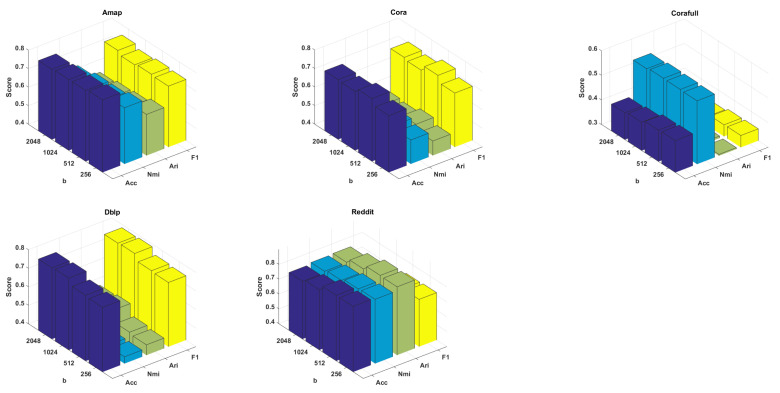
The sensitivity of our model to the batch size.

In Figure 3, we show how the performance varied with changes in the batch size within the range of {256,512,1024,2048}. From this figure, we can see that the performance fluctuation on the Amap, Cora, and Corafull datasets was not sensitive to changes in the batch size. However, a larger batch size enhanced clustering performance on Dblp; when the batch size was 1024, the clustering achieved the best results, whereas on Reddit, a smaller batch size was more beneficial for representation learning. This is because Dblp aggregated the first-order neighborhood for its representation, whereas Reddit aggregated the fifth-order neighborhood. A larger batch size facilitated the reduction of redundant relationships in Dblp but increased the risk of reducing useful relationships in Reddit.

**Figure 4:**
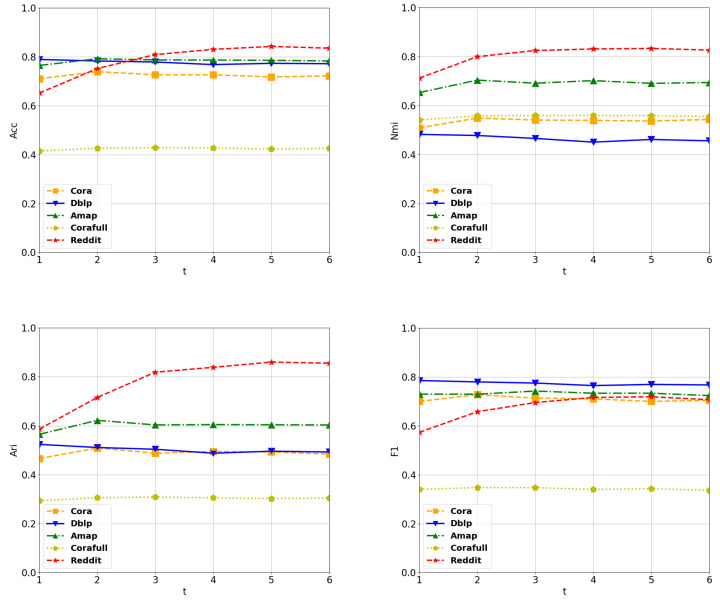
The sensitivity of our model to the power of smoothing.

In Figure 4, we illustrate how the performance varied with changes in the Laplacian smoothing power. From this figure, we can see that the ACC stabilized when the power reached 2, except for the Reddit dataset. On Reddit, the model achieved its best performance when *t* was equal to 5, and it maintained stability within the range of [3, 6]. In summary, our model demonstrated low sensitivity to these two hyperparameters, even when they varied within considerable ranges.

### 4.8. Visualization Analysis

**Figure 5:**
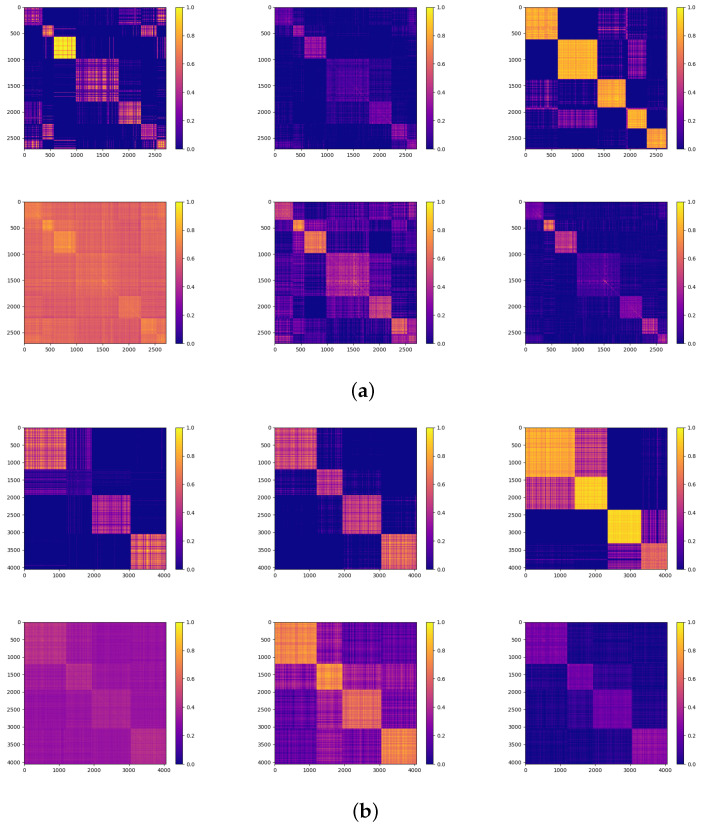
Two groups of similarity matrices with labels: (**a**) Cora, (**b**) Dblp. From top-left to bottom-right, the methods depicted are GAE, DFCN, AGCN, AutoSSL, Sublime, and our proposed method. The color scale ranges from 0 to 1, where brighter colors indicate higher similarity between corresponding nodes. A diagonal block denotes a cluster. The quality of the representation can be assessed from 2 perspectives: (1) whether the number of diagonal blocks equals the number of real clusters, and (2) whether the diagonal blocks can be easily recognized. Considering these criteria, our model can learn representations of the highest quality.

To demonstrate the effectiveness of our model in the clustering task, we illustrate a series of similarity matrices in Figure 5, showing the quality of the learned representations in each cluster. In Figure 5, we can observe that our model outperformed the other methods with respect to both the number of clusters and the clarity of the clustering structure.

## 5. Conclusions

In this paper, we propose GCHCL, a high-order contrastive learning method for graph clustering without manual augmentation. We contrast two high-order structures, constructed using two different Laplacian smoothing methods, to reveal the nodes’ similarity at the structural level, and we align the high-order structures to force the corresponding embeddings to map into the same space. After building a contrastive structure using the high-order structures, we perform contrastive learning by aligning the contrastive structure with an identity matrix. In this way, our model can naturally learn the clustering-friendly representations. Extensive experiments on datasets of various scales validate the effectiveness of the proposed model.

## Figures and Tables

**Table 1:** Notations used.

Notation	Meaning
$X\in R^{n\times f}$	Feature matrix
$\hat{X}\in R^{n\times f}$	Smoothed feature matrix
$X^{b}\in R^{b\times f}$	Sampled Features
$A\in R^{n\times n}$	Given adjacency matrix
$D\in R^{n\times n}$	Degree matrix
$Z\in R^{b\times d}$	Output of encoder
$\hat{Z}\in R^{b\times d}$	Normalized output of encoder
${\hat{Z}}_{f}\in R^{b\times d}$	Fused embeddings
$S\in R^{b\times b}$	First-order similarity matrix
$\hat{S}\in R^{b\times b}$	Second-order similarity matrix
$\tilde{S}\in R^{b\times b}$	Contrastive similarity matrix
$I\in R^{b\times b}$	Identity matrix

**Table 2:** Benchmark datasets.

Dataset	Nodes	Dimensions	Clusters	Edges	Scale
Cora	3327	3703	6	4732	small
Dblp	4058	334	4	7056	small
Amap	7650	745	8	119,081	small
Corafull	19,793	8710	70	63,421	medium
Reddit	232,965	602	41	23,213,838	large

**Table 3:** Details of hyperparameters.

Dataset	*b*	*t*	*r*
Cora	512	3	0.005
Dblp	1024	1	0.05
Amap	256	2	0.0001
Corafull	512	3	0.01
Reddit	256	5	0.05

**Table 4:** Clustering results (%) on Cora.

Method	ACC	NMI	ARI	F1
K-means	40.25±0.47	25.08±0.39	15.35±0.33	40.62±0.20
GAE	59.03±2.31	46.83±1.64	38.20±1.15	56.09±2.27
GVAE	34.37±0.74	13.41±0.36	9.12±0.42	32.59±0.69
MGAE	68.06±2.17	48.92±1.99	43.61±1.56	53.12±2.16
ARGE	64.0±0.71	44.9±0.36	35.2±0.44	61.9±1.27
ARVGE	63.8±1.58	45.0±0.65	37.4±0.80	62.7±0.76
DAEGC	66.42±1.26	48.00±0.75	42.21±1.43	63.93±1.76
SDCN	47.03±2.43	25.54±1.92	20.05±1.46	40.46±3.44
AGCN	60.56±1.33	43.59±1.81	35.46±2.35	49.76±1.34
DFCN	36.33±0.49	19.36±0.87	4.67±2.10	26.16±0.50
SCAGC	26.25±0.25	12.36±0.10	14.32±0.11	30.20±0.24
GDCL	70.83±0.47	**56.30**± 0.36	48.05±0.72	52.88±0.97
MVGRL	70.47±3.70	55.57±1.54	48.70±3.94	67.15±1.86
AutoSSL	63.81±0.57	47.62±0.45	38.92±0.77	56.42±0.21
Sublime	71.30±1.27	54.20±0.97	**50.30**± 0.77	63.50±1.26
Ours	**72.46** ±1.89	54.57±1.39	49.75±2.56	**70.89** ±2.03

**Table 5:** Clustering results (%) on Dblp.

Method	ACC	NMI	ARI	F1
K-means	38.35±0.67	10.99±0.47	6.68±0.33	32.10±0.57
GAE	53.42±2.21	29.29±1.13	16.83±1.63	54.90±1.58
GVAE	53.06±0.17	28.87±0.43	16.65±0.11	54.34±0.29
MGAE	74.49±1.85	41.67±1.23	45.81±2.11	59.67±1.67
ARGE	61.94±0.41	25.63±1.03	23.91±0.81	60.57±0.72
ARVGE	64.44±0.56	30.21±0.62	26.21±0.85	64.32±1.02
DAEGC	62.05±0.48	32.49±0.45	21.03±0.52	61.75±0.67
SDCN	68.05±1.81	39.50±1.34	39.15±2.01	67.71±1.51
AGCN	73.26±0.37	39.68±0.42	42.49±0.31	72.80±0.56
DFCN	76.00±0.82	43.7±1.14	47.00±1.52	75.70±0.81
SCAGC	47.55±1.21	45.99±0.34	12.00±0.30	11.18±1.22
GDCL	39.44±0.55	12.88±1.67	11.72±2.12	10.06±0.55
MVGRL	44.91±1.10	18.75±0.65	11.14±0.50	44.80 ±0.88
AutoSSL	40.52±1.50	12.63±0.72	5.41±0.66	37.78±1.48
Sublime	56.80±0.44	27.25±0.97	19.17±0.74	51.05±0.44
Ours	**77.55** ±0.85	**46.81** ±0.82	**49.71** ±1.56	**77.33** ±0.79

**Table 6:** Clustering results (%) on Amap.

Method	ACC	NMI	ARI	F1
K-means	27.22±0.76	13.23±1.33	5.50±0.44	23.96±0.51
GAE	71.57±2.48	62.13±2.79	48.82±4.57	68.08±1.76
VGAE	74.26±3.63	66.01±3.40	56.24±4.66	70.38±2.98
MGAE	70.42±2.56	63.30±2.33	53.46±4.36	60.35±1.69
ARGE	69.28±2.30	58.36±2.76	44.18±4.41	64.30±1.95
ARVGE	61.46±2.71	53.25±1.91	38.44±4.69	58.50±1.70
DAEGC	76.44±0.01	65.57±0.03	59.39±0.02	69.97±0.02
SDCN	53.44±0.81	44.85±0.83	31.21±1.23	50.66±1.49
AGCN	58.53±2.34	51.76±2.28	41.15±3.01	43.68±3.30
DFCN	76.88±0.23	69.21±1.21	58.98±0.74	71.58±0.31
SCAGC	42.16±0.15	21.86±0.22	17.76±0.32	31.87±0.15
GDCL	43.75±0.78	37.32±0.28	21.57±0.51	38.37±0.29
MVGRL	45.19±2.21	36.89±2.75	18.79±3.10	39.65±4.76
AutoSSL	54.55±0.97	48.56±0.71	26.87±0.34	54.47±0.83
Sublime	52.73±1.46	49.62±2.33	33.15±3.15	41.81±1.84
Ours	**79.18** ±1.06	**70.37** ±1.38	**62.22** ±1.84	**72.93** ±1.49

**Table 7:** Clustering results (%) on Corafull.

Method	ACC	NMI	ARI	F1
K-means	16.62±0.77	22.24±0.69	1.94±0.87	7.75±0.67
GAE	29.06±0.81	45.82±0.75	17.84±0.86	25.95±0.75
VGAE	32.66±1.29	47.38±1.59	20.01±1.38	29.06±1.15
MGAE	OOM	OOM	OOM	OOM
ARGE	22.07±0.43	41.28±0.25	12.38±0.24	18.85±0.41
ARVGE	29.57±0.59	48.77±0.44	18.80±0.57	25.43±0.62
DAEGC	34.35±1.00	49.16±0.73	22.60±0.47	26.96±1.33
SDCN	26.67±0.40	37.38±0.39	13.63±0.27	22.14±0.43
AGCN	OOM	OOM	OOM	OOM
DFCN	37.51±0.81	51.30±0.41	24.46±0.48	31.22±0.87
SCAGC	OOM	OOM	OOM	OOM
GDCL	OOM	OOM	OOM	OOM
MVGRL	31.52±2.95	48.99±3.95	19.11±2.63	26.51±2.87
AutoSSL	36.67±0.79	52.92±0.62	24.61±0.54	31.47±0.85
Sublime	OOM	OOM	OOM	OOM
Ours	**42.80** ±0.83	**55.93** ±0.30	**30.85** ±0.84	**34.72** ±0.93

**Table 8:** Clustering results (%) on Reddit.

Method	ACC	NMI	ARI	F1
K-means	9.79±0.05	9.61±0.07	3.07±0.05	6.96±0.04
GAE	OOM	OOM	OOM	OOM
VGAE	OOM	OOM	OOM	OOM
MGAE	OOM	OOM	OOM	OOM
ARGE	OOM	OOM	OOM	OOM
ARVGE	OOM	OOM	OOM	OOM
DAEGC	OOM	OOM	OOM	OOM
SDCN	OOM	OOM	OOM	OOM
AGCN	OOM	OOM	OOM	OOM
DFCN	OOM	OOM	OOM	OOM
SCAGC	OOM	OOM	OOM	OOM
GDCL	OOM	OOM	OOM	OOM
MVGRL	OOM	OOM	OOM	OOM
AutoSSL	OOM	OOM	OOM	OOM
Sublime	OOM	OOM	OOM	OOM
Ours	**81.92** ±0.74	**82.11** ±0.27	**84.20** ±1.26	**68.21** ±1.97

**Table 9:** Performance comparison of first-order contrastive learning and second-order contrastive learning.

Dataset	View	ACC	NMI	ARI	F1
Cora	first-order	49.77±5.21	35.64±4.69	23.11±5.08	51.82±5.89
	second-order	72.46±1.89	54.57±1.39	49.75±2.56	70.89±2.03
Dblp	first-order	69.63±6.15	39.98±4.27	40.04±6.60	69.51±6.11
	second-order	77.55±0.85	46.81±0.82	49.71±1.56	77.33±0.79
Amap	first-order	77.00±1.58	67.78±2.39	58.80±4.01	70.20±2.38
	second-order	79.18±1.06	70.37±1.38	62.22±1.84	72.93±1.49
Corafull	first-order	39.29±0.95	54.03±0.50	25.03±1.07	33.38±1.28
	second-order	42.80±0.83	55.93±0.30	30.85±0.84	34.72±0.93
Reddit	first-order	70.68±1.30	77.04±0.35	68.37±1.38	57.01±2.54
	second-order	81.92±0.74	82.11±0.27	84.20±1.26	68.21±1.97

**Table 10:** The effectiveness of each component in our model. SA denotes second-order structure alignment, and CL denotes second-order contrastive learning.

Dataset	Module	ACC	NMI	ARI	F1
Cora	w/o CL	62.50±2.68	44.38±2.43	36.94±3.03	54.19±4.00
	w/o SA	72.10±1.87	54.18±1.46	48.77±2.41	71.09±2.00
	both	72.46±1.89	54.57±1.39	49.75±2.56	70.89±2.03
Dblp	w/o CL	50.01±2.43	19.94±3.18	19.15±3.03	45.91±5.07
	w/o SA	77.08±2.40	46.21±2.48	49.63±3.55	76.63±2.42
	both	77.55±0.85	46.81±0.82	49.71±1.56	77.33±0.79
Amap	w/o CL	65.41±3.17	55.63±3.71	44.86±3.50	55.15±3.60
	w/o SA	78.37±0.92	69.49±1.45	60.29±1.87	72.71±1.83
	both	79.18±1.06	70.37±1.38	62.22±1.84	72.93±1.49
Corafull	w/o CL	34.08±1.19	49.18±0.86	20.46±1.25	25.29±1.13
	w/o SA	42.48±0.84	56.00±0.19	30.63±0.87	34.39±0.68
	both	42.80±0.83	55.93±0.30	30.85±0.84	34.72±0.93
Reddit	w/o CL	30.51±1.23	45.45±0.68	21.75±0.98	23.72±0.48
	w/o SA	37.64±1.04	54.48±0.84	31.06±0.73	28.88±1.16
	both	81.92±0.74	82.11±0.27	84.20±1.26	68.21±1.97

## Data Availability

Data available in a publicly accessible repository that does not issue DOIs Publicly available datasets were analyzed in this study. This data can be found here: Available online: https://github.com/yueliu1999/Awesome-Deep-Graph-Clustering (accessed on 8 May 2012).

## Appendix A

### Appendix A.1

The settings for the models can be found in the following references:

- GAE and VGAE [18]
- MGAE [12]
- ARGE and ARVGE [14]
- DAEGC [13]
- SDCN [24]
- AGCN [25]
- DFCN [16]
- SCAGC [20]
- GDCL [28]
- MVGRL [19]
- AutoSSL [31]
- Sublime [26]

### Appendix A.2

**Table A1:** Definitions of acronyms.

Acronym	Definition
AFGRL	Augmentation-Free Self-Supervised Learning on Graphs
AGC	Attributed Graph Clustering via Adaptive Graph Convolution
AGCN	Attention-Driven Graph Clustering Network
AGE	Adaptive Graph Encoder
AGAE	Adversarial Graph Autoencoder
ARGV	Adversarially Regularized Graph Autoencoder for Graph Embedding
AutoSSL	Automated Self-Supervised Learning for Graphs
CommDGI	Community Detection-Oriented Deep Graph Infomax
DAEGC	Deep Attentional Embedded Graph Clustering
DCRN	Dual Correlation Reduction Network
DFCN	Deep Fusion Clustering Network
DGI	Deep Graph Infomax
GAE	Graph Autoencoder
GCN	Graph Convolutional Network
GDCL	Graph Debiased Contrastive Learning with Joint Representation Clustering
GNN	Graph Neural Network
MGAE	Marginalized Graph Autoencoder
MVGRL	Contrastive Multi-View Representation Learning on Graphs
SAIL	Self-Augmented Graph Contrastive Learning
SCAGC	Self-Consistent Contrastive Attributed Graph Clustering with Pseudo-Label Prompt
SDCN	Structural Deep Clustering Network
Sublime	Structure Bootstrapping Contrastive Learning Framework
